# Editorial: Glyco-Tools to Crack Unsolved Biomedical Needs

**DOI:** 10.3389/fchem.2021.789839

**Published:** 2021-11-18

**Authors:** Filipa Marcelo, Cristina Nativi, Laura Russo, Alba Silipo, Trinidad Velasco-Torrijos

**Affiliations:** ^1^ Associate Laboratory i4HB–Institute for Health and Bioeconomy, NOVA School of Science and Technology, NOVA University Lisbon, Caparica, Portugal; ^2^ UCIBIO–Applied Molecular Biosciences Unit, Department of Chemistry / Department of Life Sciences, NOVA School of Science and Technology, NOVA University Lisbon, Caparica, Portugal; ^3^ Department of Chemistry, University of Florence, Sesto Fiorentino (FI), Italy; ^4^ Department of Biotechnology and Biosciences, University of Milano-Bicocca, Milan, Italy; ^5^ CÚRAM, SFI Research Centre for Medical Devices, National University of Ireland Galway, Galway, Ireland; ^6^ Department of Chemical Sciences, Complesso Universitario Monte S. Angelo, University of Naples Federico II, Naples, Italy; ^7^ Department of Chemistry, Maynooth University, Co. Kildare, Ireland

**Keywords:** glycans, glycomimetics, glycan binding proteins, glyconanoparticles, glycan protein interactions, glycoprobes, glycotools

**Editorial on the Research Topic**

**Glyco-Tools to Crack Unsolved Biomedical Needs**

The last advances in glycosciences have highlighted the multiple roles of glycans in complex biological systems. We are indeed now in the glycome era which, along with the genome and proteome, is essential for understanding and dissecting the complexity of cells. The improvement of structural and synthetic approaches, including microfluidic and enzymatic strategies, as well as the development of high-throughput glycan microarray technology, give us nowadays a unique opportunity to elucidate the role of glycans in distinct biological contexts (Smith and Bertozzi, 2021). Recently, glycoconjugate RNA molecules (glycoRNAs) have been identified on cell surface by Flynn et al., adding a new player into the glyco signature atlas (Flynn et al., 2021). Glycans are dynamic structures, and their composition and distribution rely on individual’s ages, diseases and diet, making them precious targets for the development of personalized theragnostic strategies. None the less, the involvement of glycans in human health and disease is more complex than we have anticipated, in fact glycans are strongly engaged in cell fate, control, signalling, and extracellular communications. In this perspective, glycoscience represents an enormous inspiration source for the design of advanced diagnostic systems and for the development of biotechnological, new generation of therapeutic treatments.

In this Editorial firefront scientific studies are described showing the multidisciplinary nature of glycoscience. Metabolic glycoengineering (MGE) is one of the most impressive techniques employable to clarify the roles of glycans. MGE has been developed in the late 1980s and today its application is showing terrific impact in both diagnostics and therapeutics (Agatemor et al., 2019). This topic is reviewed by Haiber et al., with exhaustive examples highlighting electron-demand Diels-Alder bioortogonal reactions as a cell-friendly ligation strategy suitable for healthcare applications.

Lectins are glycan binding proteins (GBP) and one of the main players for the biological relevance of glycans. Lectins are important targets due to their role in cell signalling, cell migration and differentiation or in concerted processes like angiogenesis (Johannes et al., 2018). They are mediators of pathological phenomena like cancer, inflammation and infection, playing a fundamental role in the maintenance of physiological conditions or in the unbalanced pathological cascade. Quintana et al. provide new insights on A and B histo blood group antigens binding to Galectin-4 (Gal-4), allowing the identification of interactions and their structure-based rationale thanks to the concerted employment of NMR, isothermal titration calorimetry, and molecular modelling.

Glycans are also active players involved in microbial recognition and infection. Like in mammalians biological pathways, also in microbial populations the interactions between bacteria or viruses and hosts are mediated by carbohydrate–proteins interactions. Marchetti et al. describe the recognition of host sialoglycans by hemagglutinin-neuraminidase isolated from SBL-1 strain of Mumps Viruses by concerted application of NMR, computational studies, and fluorescence assays. They also exploit a N-acetylneuraminic acid-derived thiotrisaccharide to target the viral protein.

Glycomimetics able to act as GBP inhibitors, allow to maximise the information on a large plethora of GBP involved in the maintenance of physiological processes or responsible for pathological phenomena. With this aim Weiss et al. present the synthesis and the evaluation of the activity of molecules able to bind O-GlcNAc transferase, enzyme involved in the transfer of O-GlcNAc to proteins. Thanks to a library of 2-oxo-1,2-dihydroquinoline-4-carboxamide derivatives, and the employment of different evaluation tests, interestingly false positive data obtained from commercial UDP-Glo™ glycosyltransferase assay were highlighted.

Glycans and glycomimetics can be also exploited when conjugated to macromolecular systems or nanomaterials to mimic the known multivalent behaviour of glycoconjugate proteins expressed in mammalian, microbial or vegetable biological systems. Hernando et al. provide a wide overview of carbohydrate-coated nanoparticles, overlying their use in biomedicine, including the detection of proteins or key interactors of viruses and bacteria or cancer cells. Colloidal glyconanoparticles and quantum dot have been taken into consideration, providing insights on the unresolved challenges and the future efforts needed to overcome the current limitations. Further potential applications of glyconanoparticles are reviewed by Kim et al., including the study of the interactions occurring between solid and lipid nanoparticle functionalized with glycans and receptor expressed on cell surfaces.

Polysaccharides expressed at cellular and extracellular level are also employed to develop glycan-based tools for biomedical application. Their physical and biological properties, exploited at extracellular level in tissues and organs, make them perfect scaffolds to maximise the performances of implantable medical devices. Lepedda et al. present a critical analysis of the employment of glycosaminoglycans (GAGs) as signalling molecules able to induce artery formation and, in general, their potential in the development of biomaterials for vascular regeneration.

In conclusion, the research topics collected in this Research Topic perfectly reflect the multifaced expertise which characterizes the glycoscience field and which are fundamental to path the way to future ambitious challenges in biomedical field.
